# EnsembleAge Clock: A Reliable, Robust and Convenient Epigenetic Age Prediction Service for Promoting Healthy Aging

**DOI:** 10.1093/geroni/igaf122.4316

**Published:** 2025-12-31

**Authors:** Hayan Lee, Akshay Anand, Yash Agarwal, Tanisha Gupta, Jason Lin, Mirna Ghemrawi, Glenn Gerhard

**Affiliations:** Fox Chase Cancer Center, Philadelphia, Pennsylvania, United States; University of California, Berkeley; University of California, Los Angeles, Los Angeles, California, United States; Georgia Institute of Technology, Atlanta, Georgia, United States; Brown University, Providence, Rhode Island, United States; The Center for Forensic Science Research and Education, Horsham, Pennsylvania, United States; Temple University Health System, Philadelphia, Pennsylvania, United States

## Abstract

Age is a major risk factor for various diseases, such as cancer, cardiovascular conditions, and neurodegenerative diseases. However, chronological age, the simple number of years one has lived and an unmodifiable risk factor, does not capture individual health differences, prompting the development of methods to accurately estimate biological age, modifiable by healthy habits, instead of relying on chronological age. One of the major molecular approaches exploits DNA methylation (DNAm), an essential epigenetic modifier for regulating gene expression, cell differentiation, and aging. DNAm-based aging clocks have been developed to predict biological age, but the prediction is highly dependent on training data, including assay technologies. To address these clocks’ high variance, we present EnsembleAge Clocks, leveraging previously developed DNAm clocks, harnessing the strengths of each methylation age clock, smoothing out individual variances, and providing a more robust estimation of biological age. We validated our EnsembleAge Clock models using DNA methylation data from nine organs in the GTEx dataset. Our EnsembleNaive clock model achieved the lowest median absolute error (MeAE) of 4.04 years in whole blood. The EnsembleLR model demonstrated the lowest MeAE of 6.35 years across multiple tissues, including breast, lung, muscle, ovary, prostate, testis, and colon. Our EnsembleAge clock models are also implemented as a web service (https://ensemble.epiclock.app), allowing users to conveniently upload their DNA methylation data and receive predictions of their biological age, empowering individuals to track their epigenetic age over time and promoting healthy aging with a beneficial lifestyle.

